# 358. Differences in antibiotic usage patterns in Korean hospitals according to the appropriateness of surgical prophylactic antibiotic use: a Nationwide study

**DOI:** 10.1093/ofid/ofad500.429

**Published:** 2023-11-27

**Authors:** Choseok Yoon, Jihye Shin, Dong-Sook Kim, Jungmi Chae, Sang-Heon Yoon, Yeseul Kim, wooyoung Jang, Jinnam Kim, Bongyoung Kim

**Affiliations:** Hanyang University Seoul Hosptial, Seoul, Seoul-t'ukpyolsi, Republic of Korea; Department of Research, Health Insurance Review & Assessment Service (HIRA), Wonju, South Korea, Seoul, Seoul-t'ukpyolsi, Republic of Korea; Department of Health Administration, Kongju National University, Kongju, Ch'ungch'ong-namdo, Republic of Korea; Department of Research, Health Insurance Review & Assessment Service (HIRA), Wonju, South Korea, Seoul, Seoul-t'ukpyolsi, Republic of Korea; Department of Research, Health Insurance Review & Assessment Service (HIRA), Wonju, South Korea, Seoul, Seoul-t'ukpyolsi, Republic of Korea; Department of Research, Health Insurance Review & Assessment Service (HIRA), Wonju, South Korea, Seoul, Seoul-t'ukpyolsi, Republic of Korea; Hanyang University College of Medicine, Seoul, Seoul-t'ukpyolsi, Republic of Korea; Hanyang University Seoul Hosptial, Seoul, Seoul-t'ukpyolsi, Republic of Korea; Department of Internal Medicine, Hanyang University College of Medicine, Seongdong-gu, Seoul-t'ukpyolsi, Republic of Korea

## Abstract

**Background:**

Since 2007, the Health Insurance Review and Assessment Service (HIRA) has been conducting an appropriateness evaluation of prophylactic surgical antibiotic use for all hospitals in Korea. Each of the hospitals were divided into 5 grades based on their evaluation results. The purpose of this study is to analyze the differences in antibiotic usage patterns in Korean hospitals based on the appropriateness of surgical prophylactic antibiotic use.

**Methods:**

We analyzed all antibiotics prescribed from hospitals included in the 9th appropriateness evaluation of surgical prophylactic antibiotic use from January 2020 to December 2021, using National Health Insurance claims data submitted to the HIRA. Antibiotic usage was calculated as Days of Therapy (DOT) and then adjusted to 1,000 patient-days. We compared antibiotic usage patterns with a surgical prophylactic antibiotic usage evaluation grade 1 or 2 hospitals and those with other grades hospitals. In addition, we analyzed the factors associated with grade 1 using logistic regression analysis.

**Results:**

Among a total of 338 hospitals, 198 (58.6%) of hospitals received grade 1 or 2. The total antibiotic usage (253.4 vs. 169.6, *P* < 0.001) and the usage of antibiotics predominantly used for resistant gram-positive bacteria (11.0 vs. 8.2, *P* < 0.001) were higher in grade 1-2 hospitals than in other hospitals, while the usage of broad-spectrum antibiotics mainly used for hospital-onset infections (32.1 vs. 40.3, *P*=0.043) and Narrow-spectrum beta-lactam antibiotics (135.8 vs. 165.4, *P*=0.002) were lower in grade 1-2 hospitals than in other hospitals. Logistic regression analysis showed that factors associated with grade 1 were the Charlson's comorbidity index of patients (OR 3.59, 95% CI 1.61-8.03), the proportion of cancer patients (OR 0.98, 95% CI 0.98-0.99), and the number of infectious disease specialists (OR 1.10, 95% CI 1.07-1.14).

**Figure:**
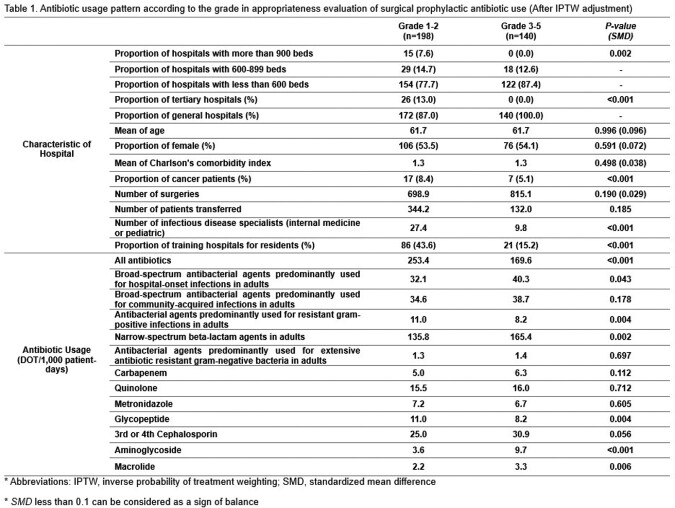

**Figure:**
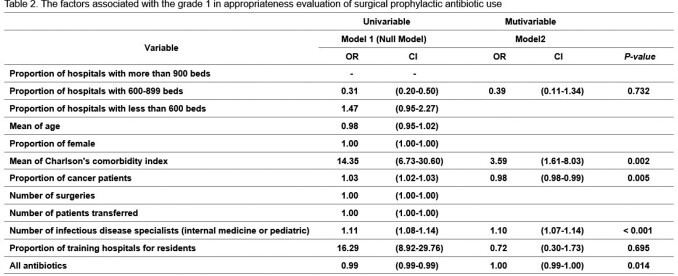

**Conclusion:**

The overall use of antibiotics and the use of antibiotics mainly used for resistant gram-positive bacteria were higher in grade 1-2 hospitals than in other hospitals; the use of broad-spectrum antibiotics commonly used for hospital-acquired infections and narrow-spectrum beta-lactam antibiotics were lower in grade 1-2 hospitals than in other hospitals.

**Disclosures:**

**All Authors**: No reported disclosures

